# Ovine conceptuses express phospholipase inhibitory genes on days 14–15 of pregnancy, interacting with IFNT pathways

**DOI:** 10.1530/REP-24-0286

**Published:** 2025-01-11

**Authors:** Yuta Matsuno, Kazuya Kusama, Koji Kimura, Kazuhiko Imakawa

**Affiliations:** ^1^ Laboratory of Molecular Reproduction, Research Institute of Agriculture, Tokai University, Kumamoto, Japan; ^2^ Department of Endocrine Pharmacology, Tokyo University of Pharmacy and Life Sciences, Hachioji, Tokyo, Japan; ^3^ Graduate School of Environmental, Life and natural science and technology, Okayama University, Okayama, Japan

**Keywords:** sheep, conceptus, uncharacterized genes, phospholipase inhibitor

## Abstract

**In brief:**

Ovine conceptuses highly express phospholipase inhibitory genes just before the conceptus attachment period. Phospholipase inhibitors could synergistically work with the interferon pathway on the endometrium.

**Abstract:**

In mammals, various molecules are involved in the biochemical interaction between the conceptus and endometrium for pregnancy recognition and establishment. In ruminants, interferon tau (IFNT) is the pregnancy recognition factor; however, IFNT alone does not explain corpus luteum maintenance. Although data on factors expressed during implantation have been accumulated, we hypothesized that the conceptus produces additional uncharacterized molecules during the period of conceptus attachment. This study aimed to identify new conceptus secretory proteins involved in the biochemical interaction between the conceptus and endometrium in sheep. We analyzed RNA-sequence data of ovine conceptuses from pregnant animals on days 12, 14, 15, 16, 17, 19, 20 and 21. To identify novel secretory proteins, we focused on highly expressed but uncharacterized genes and performed in silico protein function analysis, identifying genes encoding phospholipase inhibitory proteins expressed on days 14 and 15. Recombinant proteins from these genes were produced, and the effects on cultured bovine endometrial epithelial cells (EECs) and stromal cells (STRs) were analyzed by RNA-sequence analysis. Differentially expressed gene (DEG) analysis demonstrated that the recombinant protein treatment upregulated 31 genes and downregulated 4 genes in EECs; it also upregulated 398 genes and downregulated 66 genes in STRs, including implantation-related genes, such as *ISG15*, *OAS1X*, *OAS1Y*, *PARP9*, *PARP14*, *MX1* and *PTGS2*. Gene set enrichment analysis revealed that DEGs were enriched in several implantation-related pathways, including ISG15 antivirus mechanisms. These results suggest that, in addition to numerous characterized molecules, phospholipase inhibitory protein is a new candidate molecule in enabling biochemical communication between the conceptus and endometrium.

## Introduction

Reproductive efficiency is a critical factor in the livestock industry, with significant implications for food production and agricultural sustainability. While cattle pregnancy rates declined in the late 20th century (Royal *et al.* 2000), recent fertility-focused breeding indices and programs, such as Double Ovsynch, have significantly improved pregnancy rates (Carvalho *et al.* 2018, Cole & VanRaden 2018, Lucy 2019, Guinan *et al.* 2023). Despite these efforts, nearly 50% pregnancies still result in loss during the first few weeks (Wiltbank *et al.* 2016), underscoring a continued challenge in the livestock industry. Despite extensive research contributing to our fundamental understanding of estrous cycle control, ovulation, timing of artificial insemination, *in vitro* embryo production, embryo transfer and cryopreservation, our knowledge is not sufficient to mitigate early embryonic loss in ruminants.

Sheep share conserved biological pathways governing conceptus elongation and establishment of pregnancy with cattle (Spencer *et al.* 2017, Bazer & Johnson 2024). In fact, studies on sheep have provided critical insights into the complex conceptus–endometrial interactions that are translatable to cattle. One of the key processes during early pregnancy in ruminants is the biochemical interaction between the elongating conceptus (embryo and extraembryonic tissues) and the maternal endometrium (Lonergan & Forde 2014). The maintenance of the corpus luteum, which produces progesterone to support pregnancy, is a vital step in this process (Spencer *et al.* 2007). Trophoblast cells, which form the outer layer of the elongating conceptus, play a crucial role by secreting factors that modulate the maternal environment (Imakawa *et al.* 2004).

Among extracellular factors derived from trophoblast cells, interferon tau (IFNT) has been identified as the primary pregnancy recognition factor that prevents luteal regression in ruminants (Imakawa *et al.* 1987). Since its discovery, IFNT has been extensively studied for its role in enhancing pregnancy rates (Ealy & Wooldridge 2017, Forde & Lonergan 2017, Hansen *et al.* 2017, Roberts 2017). However, IFNT alone does not fully account for the complex mechanisms governing pregnancy establishment (Imakawa *et al.* 2017). This suggests that additional trophoblast-derived factors are also important for the early pregnancy period.

Accumulated data from extensive transcriptome and proteomics analyses indicate the presence of other extracellular factors produced by trophoblast cells, including secretory proteins, extracellular proteins and extracellular vesicles, that interact with the uterine endometrium (Forde *et al.* 2011, 2012, Mathew *et al.* 2019, Sánchez *et al.* 2019, Malo Estepa *et al.* 2020, Nakamura *et al.* 2020, 2023, Matsuno *et al.* 2021). Identifying and characterizing these factors is crucial for a holistic understanding of early pregnancy and for developing strategies to improve reproductive efficiency in mammals. Despite focusing on the factors identified so far, methods to effectively prevent early embryonic loss remain unclear. It is therefore highly likely that there remain additional yet-unidentified factors crucial to the process of early pregnancy.

In this study, we aimed to identify novel conceptus-derived factors, previously uncharacterized, involved in the biochemical interaction between the conceptus and endometrium by conducting RNA sequencing analysis of the ovine conceptus during the peri-implantation period. We focused on highly expressed but uncharacterized genes that encode putative extracellular proteins by in silico analysis to uncover new, previously unknown proteins that might play critical roles in the peri-implantation period. As over 50% pregnancies are lost currently, exploring these proteins could contribute to identifying mechanisms that further improve pregnancy rates. This analysis identified three candidate genes for encoding phospholipase inhibitory proteins. We produced their recombinant proteins and evaluated their effects on cultured endometrial epithelial cells (EECs) and stromal cells (STRs) by RNA-sequencing analysis, from which the expression of implantation-related pathways and genes was affected. The results provide new insights into our understanding of conceptus factors critical for pregnancy establishment in ruminants.

## Materials and methods

All reagents were purchased from FUJIFILM Wako Pure Chemical Corporation (Japan) unless otherwise noted.

### RNA-sequence data processing and analysis

We previously reported the RNA-sequence data obtained from day 15, 17, 19 and 21 ovine conceptuses (day 0 = day of estrus) (*n* = 3–4/day) (Matsuno *et al.* 2021) and deposited them to the Data Bank of Japan (DDBJ, PRJDB11121, http://www.ddbj.nig.ac.jp). We obtained RNA-sequence data of pregnant day 12, 14, 16 and 20 ovine conceptuses (*n* = 3–5/day) (Brooks *et al.* 2016) from Gene Expression Omnibus (GEO, GSE87017, https://www.ncbi.nlm.nih.gov). The conceptuses corresponding to the periods of pre-contact (day 12), apposition (day 14), pre-attachment (day 15), on and right after attachment (days 16, 17 and 19) and post-attachment (days 20 and 21) were analyzed by integrating these data sets.

The downstream analyses were performed using RaNA-seq (Prieto & Barrios 2020) with its default parameters. In brief, the raw FASTQ files were subjected to a quality check to remove low-quality reads and adapter reads using fastp (Chen *et al.* 2018). The trimmed reads were aligned with the Oar_v3.1 ovine genome annotated with genes to generate the gene expression values in the normalized form of transcripts per million (TPM) (Wagner *et al.* 2012) using Salmon (Patro *et al.* 2017).

### Extracting the high expression uncharacterized genes in ovine conceptuses during the peri-implantation period

Based on the average TPM value of the replicates, the top 100 high-expression genes, including expressed sequence tags (ESTs) (Tugendreich *et al.* 1993), were extracted from conceptuses on each pregnant day. Among the extracted genes, the ESTs not annotated with official gene symbols were considered uncharacterized genes and subjected to further analysis.

### Predicting the protein function of the highly expressed uncharacterized genes

The amino acid sequences of translated products of the uncharacterized genes were obtained using the getSequence function of biomaRt (Durinck *et al.* 2005, 2009). The amino acid sequences were subjected to functional protein analysis using InterPro (Paysan-Lafosse *et al.* 2023; http://www.ebi.ac.uk/interpro/) to predict their biological processes, molecular functions, cellular components and protein domains. The genes classified as encoding extracellular proteins are listed in Supplemental Table 1 (see section on Supplementary materials given at the end of the article).

### Animals and tissue collection

The tissue samples used in this study were collected from whiteface crossbred ewes. The protocol for sheep experimentation had previously been reviewed and approved by the animal care committee at the University of Tennessee due to collaborative work carried out with Dr James D Godkin, affiliated with the institution at the time of the study. Animal care, estrous synchronization procedures and tissue collections were performed as described previously (Imakawa *et al.* 2002). Conceptuses were collected using the method previously described and frozen at −80 °C until use (Imakawa *et al.* 2006, Sakurai *et al.* 2012).

### RNA extraction and quantitative reverse transcription PCR analysis

Total RNA extraction was performed as reported previously (Matsuno *et al.* 2022). In brief, total RNA was extracted from frozen conceptuses at days 15, 17, 19 and 21 (*n* = 3/day) using ISOGEN2 (Cat: 311-07361; NIPPON GENE Co. Ltd, Tokyo, Japan) according to the manufacturer’s protocol.

Real-time PCR analysis was performed as reported previously (Matsuno *et al.* 2019). In brief, the extracted RNA was reverse-transcribed to cDNA using a ReverTra Ace qPCR Master Mix with gDNA Remover (Cat: FSQ-301; Toyobo, Japan) according to the manufacturer’s protocol. Real-time PCR was performed according to the manufacturer’s protocol using a THUNDERBIRD Next SYBR qPCR Mix (Cat: QPX-201; Toyobo) and a StepOnePlus real-time PCR system (Thermo Fisher Scientific). The transcript levels were normalized to the levels of a reference gene, actin beta (ACTB), using the 2^−ΔΔ^Ct method (Livak & Schmittgen 2001). Dissociation-curve analyses were performed at the end of each analysis to avoid false-positive signals. The PCR primers used are listed in Supplementary Table 2.

### Cloning

The cDNA obtained from frozen conceptuses at day 15 (*n* = 1/day) was used as a template for PCR amplification. The full-length cDNA region of LOC101108413 (transcript ID: ENSOART00000009537.1) was amplified using Quick Taq HS DyeMix (Cat: DTM-101; Toyobo) and specific primers (Supplementary Table 2) according to the manufacturer’s protocol. The products were subjected to TA-cloning using pGEM-T Easy Vector Systems (Cat: A1360; Promega K.K., Japan), and their nucleotides were Sanger sequenced at the Medical Science College Office of Tokai University.

### Production and purification of the recombinant protein

To produce the recombinant protein, the cloning DNA sequence was codon-optimized and cloned into a pcDNA3.1(+)-C-6His expression vector (GenScript Japan Inc., Japan) at BamHI/NotI sites (Supplementary data 1). The codon-optimized DNA sequence was constructed by GenScript Japan Inc. The insert sequence and the flanking sequence of the cloning site were confirmed by Sanger sequencing.

The plasmid was transfected into HEK-293 cells (human embryonic kidney 293 cells) using TransIT-LT1 (Cat: MIR2300; Mirus Bio Corporation, USA) according to the manufacturer’s protocol. After 48 h, the culture medium was collected. The recombinant protein from the culture medium was purified using a His-tagged protein purification kit (Cat: 3310; Medical & Biological Laboratories Co., Ltd, Japan) according to the manufacturer’s protocol. Protein production was confirmed by western blotting and silver staining using SDS-PAGE with mouse anti-6× histidine antibody (Cat: 66005-1-IG; Proteintech Group Inc., USA), HRP-conjugated goat anti-mouse IgG(H+L) (Cat: SA00001-1; Proteintech Group Inc.) and Silver Stain 2 Kit wako (Cat: 291-50301) according to the manufacturer’s protocol. Signals were visualized using an ImmunoStarLD kit (Cat: 290-69904) and a WSE-6200 LuminoGraph II (ATTO Corporation Inc., Japan).

### The recombinant protein treatment on bovine endometrial epithelial cells (EECs) and stromal cells (STRs)

Bovine EECs and STRs were collected as described previously (Skarzynski *et al.* 2000, Murakami *et al.* 2003, Sakai *et al.* 2022). In brief, uteri from healthy cows with no visible conceptus were obtained at a local abattoir (Okayama Meat Center and Tsuyama Meat Center) within 10–20 min of exsanguination. These uteri were immediately transported to the laboratory and submerged in ice-cold physiological saline. Estrous cycle stages were confirmed by macroscopic examination of the ovaries, with the early estrous cycle (days 2–5) uteri designated for endometrial cell isolation and culture. EECs and STRs from the bovine endometrium were enzymatically separated. Hank’s balanced salt solution containing 0.3% trypsin was used to fill the endometrial lumen and detach the EECs. After collecting the EECs, collagenase was applied to digest the intercaruncular endometrium, isolating the STRs. The collected EECs and STRs were then individually resuspended in the culture medium. The collected EECs and STRs were used in experiments within three passages. The basic culture medium used was DMEM/F-12 (Cat: 045-30285) supplemented with antibiotic-antimycotic solution (Cat: 15240–062, Thermo Fisher Scientific K.K., Japan), L-alanyl-L-glutamine solution (Cat: 016-21841), sodium pyruvate (Cat: 190-14881) and MEM Non-Essential Amino Acids (Cat: 139-15651). Isolated bovine EECs and STRs were cultured in a well of a 12-well plate (1.0 × 10^5^ cells/well) (Cat: 83.3921, Sarstedt AG&Co. KG, Germany) coated with Matrigel (Cat: 354234, Corning, USA) using the basic culture medium supplemented with 10% (v/v) fetal bovine serum at 37.5 °C with 5% CO_2_ for 24 h for attachment. Afterward, the cells were cultured in basic culture medium supplemented with ITS-G supplement (Cat: 090-06741) with or without the recombinant protein (10 ng/mL) for 48 h. Then, the cells from three wells were combined and used as one replicate for further analysis.

### RNA-sequence analysis of cultured bovine EECs and STRs treated with or without the recombinant protein

Total RNA was extracted from the cultured cells (*n* = 3 per condition) using ReliaPrep RNA Cell Miniprep System (Cat: Z6012; Promega K.K., Woods Hollow Road, Madison, USA) according to the manufacturer’s protocol. RNA integrity number (RIN) of the samples was examined using an Agilent 2100 Bioanalyzer kit (Agilent Technologies, Palo Alto, USA) and an Agilent RNA 6000 kit (Agilent Technologies) according to the manufacturer’s protocol. The RIN values were between 9.2 and 10.

Library construction, quality control and sequencing were performed by Filgen Inc. (Japan). In brief, a total of 3 μg RNA per sample was used as an input material for RNA sample preparations. mRNA was purified from total RNA using poly-T oligo-attached magnetic beads. Sequencing libraries were generated using NEBNext Ultra RNA Library Prep Kit for Illumina (New England Biolabs Japan Inc., Japan). The library was subjected to sequencing using Illumina NovaSeq 6000, and 150 base paired-end reads were generated. The sequenced reads (raw reads) were subjected to several quality checks. In this step, clean data (clean reads) were obtained by removing reads containing adapter and poly-N (N represents the undetermined bases) and low-quality reads from raw data. The filtering process was as follows: (i) remove reads containing adapters, (ii) remove reads containing *n* > 10% and (iii) remove reads containing low-quality (Q score ≤5) bases, which were over 50% of the total base. The data have been deposited in the Data Bank of Japan (DDBJ, PRJDB18558, http://www.ddbj.nig.ac.jp).

The downstream analyses were performed using RaNA-seq (Prieto & Barrios 2020) with its default parameters described above. In brief, the raw FASTQ files were subjected to quality checks to remove low-quality reads and adapter reads using fastp (Chen *et al.* 2018). The trimmed reads were aligned with the UMD3.1 bovine genome annotated with genes and transcripts to generate gene expression values in the normalized form of TPM (Wagner *et al.* 2012) using Salmon (Patro *et al.* 2017). The count number was normalized by size factor and then subjected to principal component analysis (PCA) using the plotPCA function of DESeq2 (version 1.44.0; https://bioconductor.org/packages/release/bioc/html/DESeq2.html) (Love *et al.* 2014). Significant differentially expressed genes (DEGs) were determined with an adjusted *P*-value <0.05. This is the default parameter in RaNA-seq, where DEGs are identified without fold changes. The aligned read count data were used for gene set enrichment analysis (GSEA) using RaNA-seq (Prieto & Barrios 2020).

### Statistical analysis

All experiments were performed independently and repeated at least three times. Statistical analyses were conducted using RStudio (version 2024.04.1+748; https://posit.co/products/open-source/rstudio/) (RStudio Team 2020) with R (version 4.4.0; https://www.r-project.org/) (R Core Team 2021). The Tukey–Kramer test was used for multiple comparisons. *P*-value <0.05 was considered statistically significant.

## Results

### High expression of uncharacterized genes in ovine conceptuses during peri-implantation periods and prediction of their protein function

**Figure 1:**
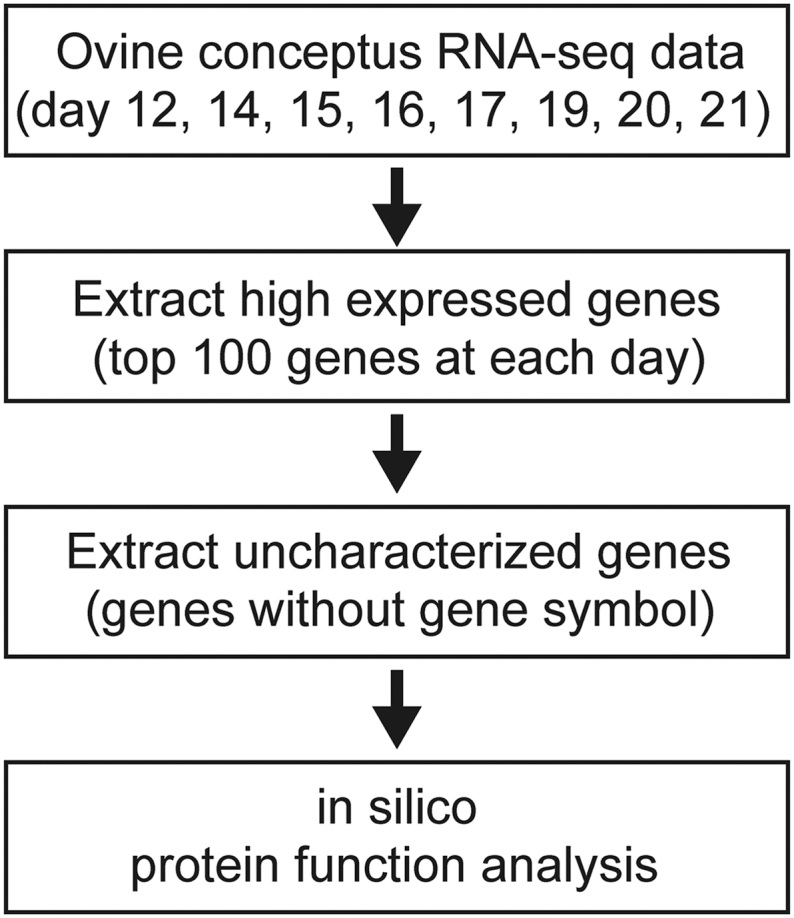
Study design to identify the highly expressed uncharacterized genes in ovine conceptuses during peri-implantation period. The RNA-sequencing data of ovine conceptuses at pregnant days 12, 14, 15, 16, 17, 19, 20 and 21 (data sets from PRJDB11121 and GSE87017) were used in this study. The top 100 expression genes based on transcripts per million value were extracted at each pregnant day. Of these genes, the genes that were not annotated with gene-symbol were subjected to *in silico* protein function analysis.

We analyzed RNA sequencing data from ovine conceptuses on days 12, 14, 15, 16, 17, 19, 20 and 21 and focused on the highly expressed genes (Fig. 1). Analysis of the top 100 expressed genes on days 12, 14, 15, 17, 19, 20 and 21 of pregnancy revealed 213 unique genes after duplicate removal; notably, 90 of these genes were without official gene symbols (Supplementary Table 3). Then, we extracted the genes without official gene symbols for further analysis and analyzed their amino acid sequences of transcribed products. In this analysis, we identified nineteen genes predicted to encode extracellular proteins, including those in the extracellular region, extracellular space, collagen-containing extracellular matrix or intermediate-density lipoprotein particle (Supplemental Table 1).

The *in silico* protein function analysis identified the biological processes and molecular functions involved in conceptus implantation, such as ‘defense response’, ‘regulation of cell population proliferation’, ‘lipid transport’, ‘lipoprotein metabolic process’ and ‘regulation of immune system process’.

In addition, novel biological processes and molecular functions were also represented in the analysis, such as ‘serine-type endopeptidase inhibitor activity’, ‘phospholipase inhibitor activity’, ‘defense response to Gram-negative bacterium’ and ‘defense response to Gram-positive bacterium’ (Supplemental Table 1). Full details of the identified biological processes and molecular functions in each gene are shown in Supplemental Table 1.

### Expression pattern of the phospholipase inhibitory genes during the peri-implantation periods

**Figure 2:**
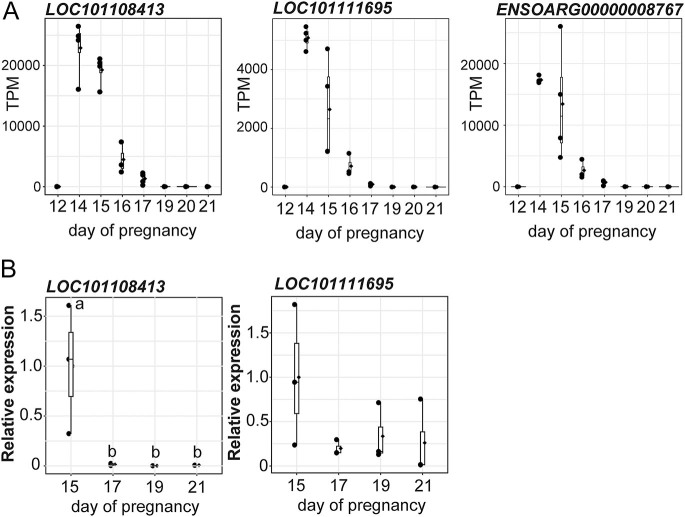
Expression of the phospholipase inhibitory genes in ovine conceptus during the peri-implantation period. (A) The expression values were shown as transcripts per million (TPM). (B) RT-qPCR analysis of transcripts encoding *LOC101108413* and *LOC101111695* in ovine conceptuses at pregnant days 15, 17, 19 and 21. RNA was extracted from frozen conceptuses (*n* = 3/day). Data relative to the value of day 15 are shown. The values represent each sample. The values with different letters (a and b) are significantly different (*P* < 0.05).

Of the various candidates, we focused on phospholipase inhibitor activity because this biological process is critically involved in the production of prostaglandin, such as prostaglandin F2 alpha (PGF2α), but is not yet characterized as a biological process associated with conceptus implantation; in addition, these genes were highly expressed just before the onset of the implantation period (Fig. 2A).

The expression patterns of the genes characterized with phospholipase inhibitor activity were analyzed as TPM values on days 12, 14, 15, 16, 17, 19, 20 and 21 of pregnancy (Fig. 2A). The results showed that *LOC101108413*, *ENSOARG00000008767* and *LOC101111695* were minimally expressed on day 12, exhibited a transient peak in expression on days 14–15 and were barely detectable from day 17 onward in the conceptus.

qRT-PCR analysis demonstrated the high expression of *LOC101108413* and *LOC101111695* in conceptuses on day 15, while the expressions were barely detectable in conceptuses on days 17 and 21 (Fig. 2B). These results are in agreement with the RNA-sequence results. Due to difficulties in designing specific primers, *ENSOARG00000008767* was not included in the qRT-PCR analysis.

**Figure 3:**
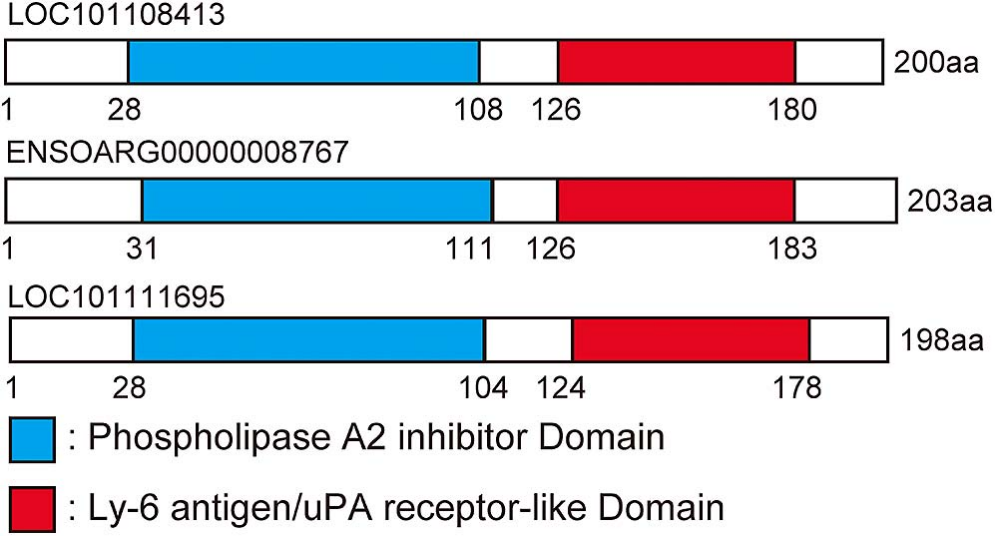
Schematic illustration of the phospholipase inhibitory gene structures. The predicted protein domains are shown in blue (Phospholipase A2 inhibitor, N-terminal domain, InterPro ID: IPR004126) and red (Ly-6 antigen/uPA receptor-like, InterPro ID: IPR016054), respectively.

The domain structures are shown in Fig. 3. These genes have a phospholipase A2 inhibitor, N-terminal domain and a Ly-6 antigen/uPA receptor-like domain (Fig. 3). The predicted domain structures of the three gene products are highly comparable.

### Effects of recombinant phospholipase inhibitory protein on cultured bovine EECs and STRs

To elucidate the function of a gene predicted to have phospholipase inhibitory activity, we generated its recombinant protein. SDS-PAGE with silver staining and western blot analysis using His-tag antibodies confirmed the successful production of the desired-sized recombinant protein (Supplemental Fig. 1).

Next, we examined the effect of the treatment of the recombinant protein on cultured bovine EECs and STRs.

**Figure 4:**
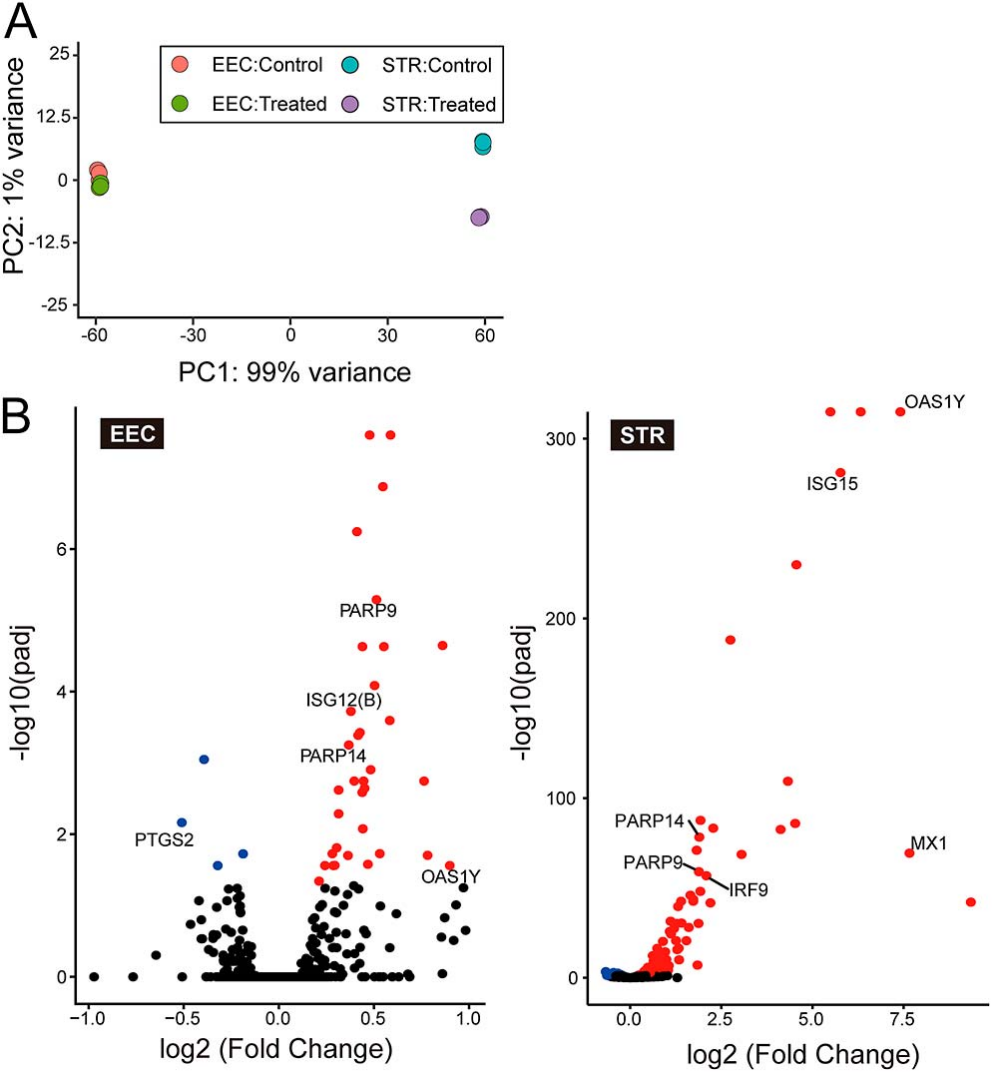
Transcript expression profiles of cultured bovine endometrial epithelial cells (EEC) and stromal cells (STRs) treated with or without the phospholipase inhibitory recombinant protein. (A) Principal component analysis of the transcripts of the cultured bovine EECs and STRs treated with or without the phospholipase inhibitory recombinant protein. (B) Volcano plot analysis of the differentially expressed transcripts. Adjusted *P*-value (padj) <0.05 was considered as significantly different. The blue and red dots represent significantly decreased and increased transcripts, respectively.

PCA showed clear branching between EECs and STRs (Fig. 4A). STRs were clearly separated between control and treated groups, while EECs were clustered considerably close to each other between control and treated groups. Analysis of DEGs revealed that the treatment of recombinant proteins on EECs resulted in the upregulation of 31 genes and the downregulation of 4 genes, while 398 genes were upregulated and 66 genes were downregulated in STRs (Fig. 4B).

**Figure 5:**
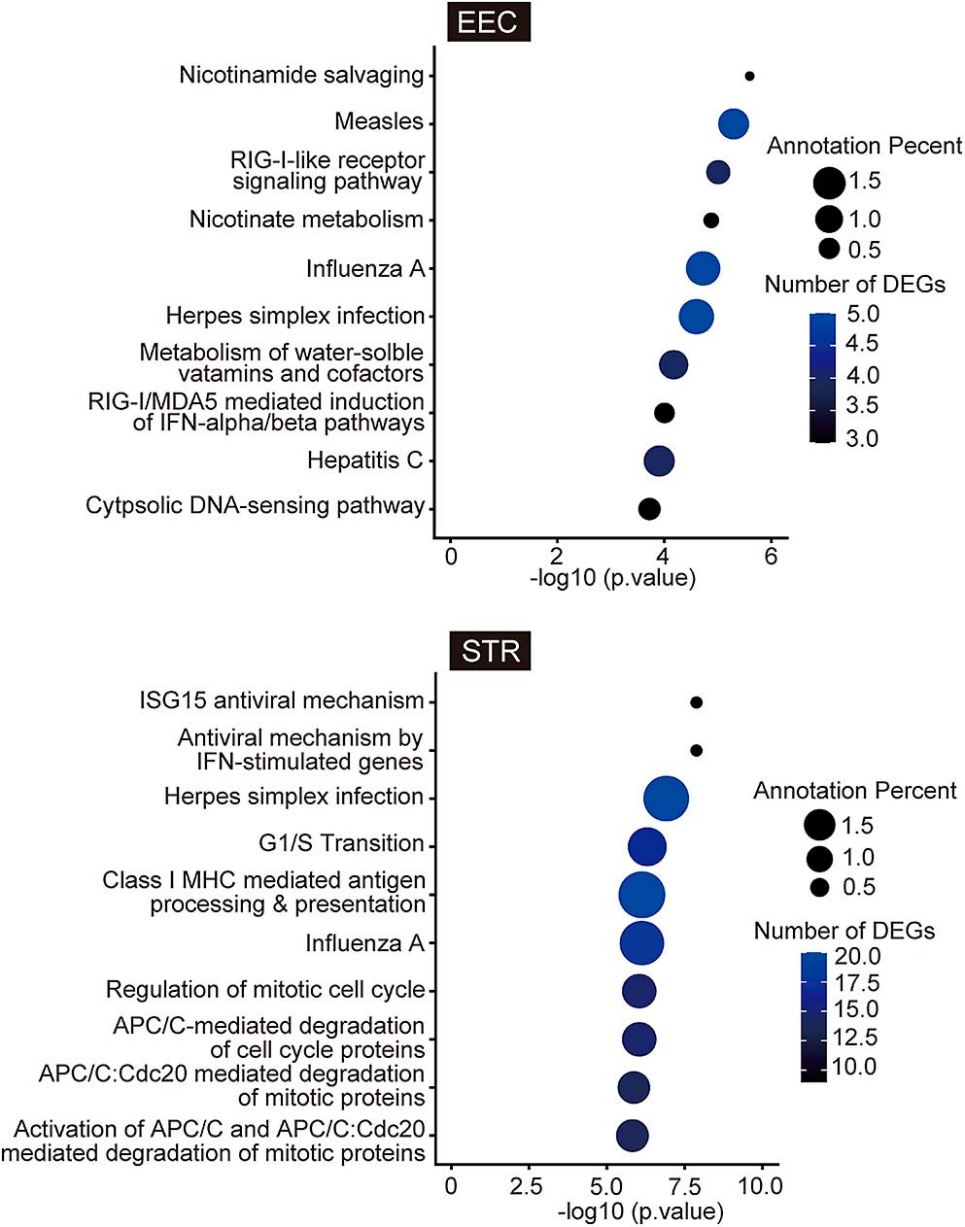
Gene set enrichment analysis of the differentially expressed genes in the cultured bovine endometrial epithelial cells (EECs) and stromal cells (STRs) treated with or without the phospholipase inhibitory recombinant protein. Enriched pathways are shown. The larger circles indicate a higher percentage of annotated genes. The color of the circles represents the number of significantly differentially expressed genes in each pathway, with blue indicating a higher number and black indicating a lower number. Details are shown in Supplemental Table 4.

To identify the enriched pathways in DEGs in EECs and STRs, we performed GSEA (Fig. 5). GSEA indicated significant enrichment in several pathways, including ‘nicotinamide salvaging’, ‘measles’, ‘RIG-I-like receptor signaling pathway’, ‘nicotinate metabolism’, ‘influenza A’, ‘herpes simplex infection’, ‘metabolism of water-soluble vitamins and cofactors’, ‘RIG-I/MDA5-mediated induction of IFN-alpha/beta pathway’, ‘hepatitis C’ and ‘cytosolic DNA-sensing pathway’ in EECs, while ‘ISG15 antiviral mechanism’, ‘antiviral mechanism by IFN-stimulated genes’, ‘G1/S transition’, ‘class I MHC-mediated antigen processing and presentation’, ‘influenza A’, ‘regulation of mitotic cell cycle’, ‘APC/C-mediated degradation of cell cycle proteins’, ‘APC/C:Cdc20-mediated degradation of mitotic proteins’ and ‘activation of APC/C and APC/C:Cdc20-mediated degradation of mitotic proteins’ in STRs. Full details of the identified pathways in DEGs in EECs and STRs are given in Supplemental Table 4.

## Discussion

In our study, we identified highly expressed but uncharacterized genes in ovine conceptuses during the peri-implantation period. Of these, genes encoding phospholipase inhibitor proteins were notably expressed at the onset of conceptus attachment.

Phospholipases hydrolyze phospholipids, producing arachidonic acid, a precursor for prostaglandin biosynthesis, crucial for regulating pregnancy (Murakami *et al.* 2020). Specifically, phospholipase A2 (PLA2) catalyzes the release of arachidonic acid from membrane phospholipids, leading to prostaglandin synthesis (Tithof *et al.* 2007, Godkin *et al.* 2008). The inhibition of prostaglandin F2 alpha (PGF2α) is necessary for pregnancy continuation in ruminants, as it suppresses the endometrial luteolytic mechanism, maintaining progesterone production by the corpus luteum (Spencer & Hansen 2015). This suppression is primarily mediated by interferon tau (IFNT), produced by the elongating conceptus, which inhibits the upregulation of oxytocin receptors in the endometrial epithelia, disrupting the pulsatile release of luteolytic PGF2α (Spencer *et al.* 2007). Previous research highlighted distinct roles of different PLA2 enzymes in regulating prostaglandin production. PLA2G6, a calcium-independent phospholipase A2, significantly influences PGF2α synthesis in bovine endometrial cells. Oxytocin-stimulated PGF2α production, mediated through PLA2G6, is inhibited by IFNT (Godkin *et al.* 2008). Moreover, the PLA2G6 inhibitor bromoenol lactone effectively abolishes oxytocin-stimulated PGF2α production, underscoring the enzyme’s specific role (Godkin *et al.* 2008). In addition, previous research identified phospholipase inhibitory proteins in extracellular vesicles from cultured ovine conceptuses (Burns *et al.* 2016). Whether phospholipase inhibitory proteins secreted by the conceptus suppress the pulsatile release of PGF2α from the endometrium requires further investigation.

PCA revealed a distinct separation between treated and control endometrial STRs, whereas EECs did not show a clear separation (Fig. 4A). This indicates more significant effects of phospholipase inhibitory proteins on STRs. Our monolayer culture likely contributed to the more pronounced gene expression changes observed, as recombinant proteins directly interacted with STRs, bypassing epithelial layers. This monolayer culture may exaggerate gene expression effects compared to *in vivo* conditions, where proteins secreted by the conceptus primarily interact with the epithelial cells in uterine lumen fluid. Future studies are needed to explore these epithelial–stromal interactions in more detail using more complex models, such as co-culture systems or uterine organoids.

Differential gene expression analysis identified significant alterations in interferon-stimulated genes, including *IRF9*, *ISG12(B)*, *ISG15*, *OAS1X*, *OAS1Y*, *PARP9*, *PARP14* and *MX1* (Bai *et al.* 2022) (Fig. 4B). In addition, the expression of *PTGS2*, the gene encoding the enzyme that converts arachidonic acid to prostaglandin H2, was significantly reduced in EECs, indicating transcriptional suppression of prostaglandin synthesis (Fig. 4B). A recent study demonstrated that the phospholipase A2 inhibitor and LY6/PLAUR domain-containing protein PINLYP regulates type I interferon innate immunity (Liu *et al.* 2022). These findings suggest a potential synergistic action between phospholipase inhibitory proteins and interferon pathways on the endometrium during the initiation of conceptus attachment in ruminants.

The results of the GSEA indicate a notable enrichment of pathways associated with viral infection, likely linked to the regulation of interferon pathways. The prominence of viral infection-related pathways suggests that the phospholipase inhibitory protein might modulate the uterine immune response, potentially enhancing interferon activity. This hypothesis is supported by the identification of interferon-stimulated genes in our differential expression analysis, suggesting a coordinated regulation of immune and reproductive processes. However, it is important to note that there could be subtle differences in receptor–ligand interactions or signaling pathway dynamics between cattle and sheep. We used bovine cells instead of ovine ones in this study because well-characterized and readily available primary cultures of bovine endometrial cells are extensively used as models for studying the conceptus–endometrium interaction. Further studies using ovine-specific cell models are warranted.

The gene encoding phospholipase inhibitory protein is expressed in testes and stomach in mice (GeneAtlas, data set: MOE430, gcrna, http://biogps.org/?full#goto=genereport&id=641361) and in a broad range of tissues and does not exhibit tissue specificity in humans (Human Protein Atlas, https://www.proteinatlas.org/ENSG00000234465-PINLYP) (Sjöstedt *et al.* 2020). CRISPR/Cas9 knockout studies have shown that mice having 7 bp deletion in exon 1 in this gene remain fertile (Miyata *et al.* 2016), while mice having 92 bp deletion in exons 1 and 2 undergo early embryonic lethality (Liu *et al.* 2022), indicating that possibly due to truncated expression, maintaining function and its role in fertility may be non-essential or compensated by other mechanisms. However, the implantation process in ruminants differs significantly from that in mice, with ruminants exhibiting a prolonged pre-attachment period and non-invasive placentation (Guillomot 2019, Johnson *et al.* 2023). In ruminants, implantation involves three main stages: pre-attachment, apposition and adhesion, leading to an epitheliochorial placenta (Guillomot 2019). Trophoblast cells in ruminants undergo morphological and functional changes, including the formation of binucleate cells that fuse with uterine epithelial cells (Spencer *et al.* 2004, Guillomot 2019, Yamada *et al.* 2022). Despite some similarities, trophoblast development and differentiation differ between ruminants and mice from early stages (Pfeffer & Pearton 2012). Therefore, to elucidate the function of the gene encoding phospholipase inhibitory protein, which is highly expressed in trophoblast cells just before conceptus attachment, it is crucial to study ruminant models, such as sheep or cattle, rather than mice. This approach will provide more relevant insights to understand the biochemical interaction between the conceptus and endometrium specific to ruminants. A search of the phospholipase inhibitory genes identified in sheep for this study using the basic local alignment search tool (BLAST) shows a top hit with the bovine phospholipase A2 inhibitor and LY6/PLAUR domain-containing gene (*PINLYP*), suggesting a high likelihood of conserved function between sheep and cattle.

In this study, we focused on phospholipase inhibitor proteins because of their predicted roles in implantation and the conceptus–endometrium interaction. However, we also identified non-phospholipase inhibitor proteins that may play significant roles in early pregnancy and conceptus signaling pathways (Supplemental Table 1). Although not analyzed in this study, they represent promising candidates for future research.

Investigations into specific genes, their expression and their product levels during early pregnancy periods utilize high-throughput techniques, such as RNA sequencing, proteome, metabolome and lipidome analysis, to identify and assess various biological molecules, such as gene expression, lipids and metabolites in ruminants. Uncharacterized genes within ESTs provide a unique opportunity to identify previously unknown regulators or modulators of implantation. We selected the ESTs that exhibited high expression levels and were predicted to encode extracellular proteins based on bioinformatic analysis, as these proteins are likely to play a role in communication between the conceptus and the endometrium. Since more than 50% pregnancies are still lost, in addition to well-characterized functional genes, targeting uncharacterized genes and their interaction with already characterized genes offer potential for discovering mechanisms that could improve implantation rates. Our approach may contribute to further understand early embryonic loss in ruminants.

## Conclusion

In conclusion, this study has unveiled novel characteristics of conceptus-derived proteins, which may potentially function extracellularly, with phospholipase inhibitory domain, highlighting a previously unrecognized mechanism during conceptus attachment period in ruminants. The identification of phospholipase inhibitory activity opens new avenues for addressing the gap in our understanding of the mitigation of early embryonic loss in ruminants.

## Supplementary materials



## Declaration of interest

The authors declare that there is no conflict of interest that could be perceived as prejudicing the impartiality of the work.

## Funding

This work was supported by Livestock Promotional Funds of Japan Racing Association (JRA), the grants from Kieikai Research Foundationhttps://doi.org/10.13039/501100012014, a grant for Young Scientists from RIKAKEN HD, a grant from The Morinaga Foundation for Health & Nutritionhttps://doi.org/10.13039/501100009583 and a Grant-in-Aid for Young Scientists (21K14965) from Japan Society for the Promotion of Science (JSPS)https://doi.org/10.13039/501100001691. This study was also supported in part by the Research Institute of Agriculture, Tokai University.

## Author contribution statement

YM performed experiments, analysis, prepared all figures and tables and wrote the original draft. K Kimura performed isolation of bovine EECs and STRs. KI conceived the study and wrote the paper. YM, K Kusama, K Kimura and KI reviewed the manuscript.
