# Supplementary figures and images for: Ovine conceptuses express phospholipase inhibitory genes on days 14–15 of pregnancy, interacting with IFNT pathways
Source: Reproduction. 2025 Jan 11;169(2):e240286. doi: 10.1530/REP-24-0286 (PMC11935640; doi:10.1530/REP-24-0286)

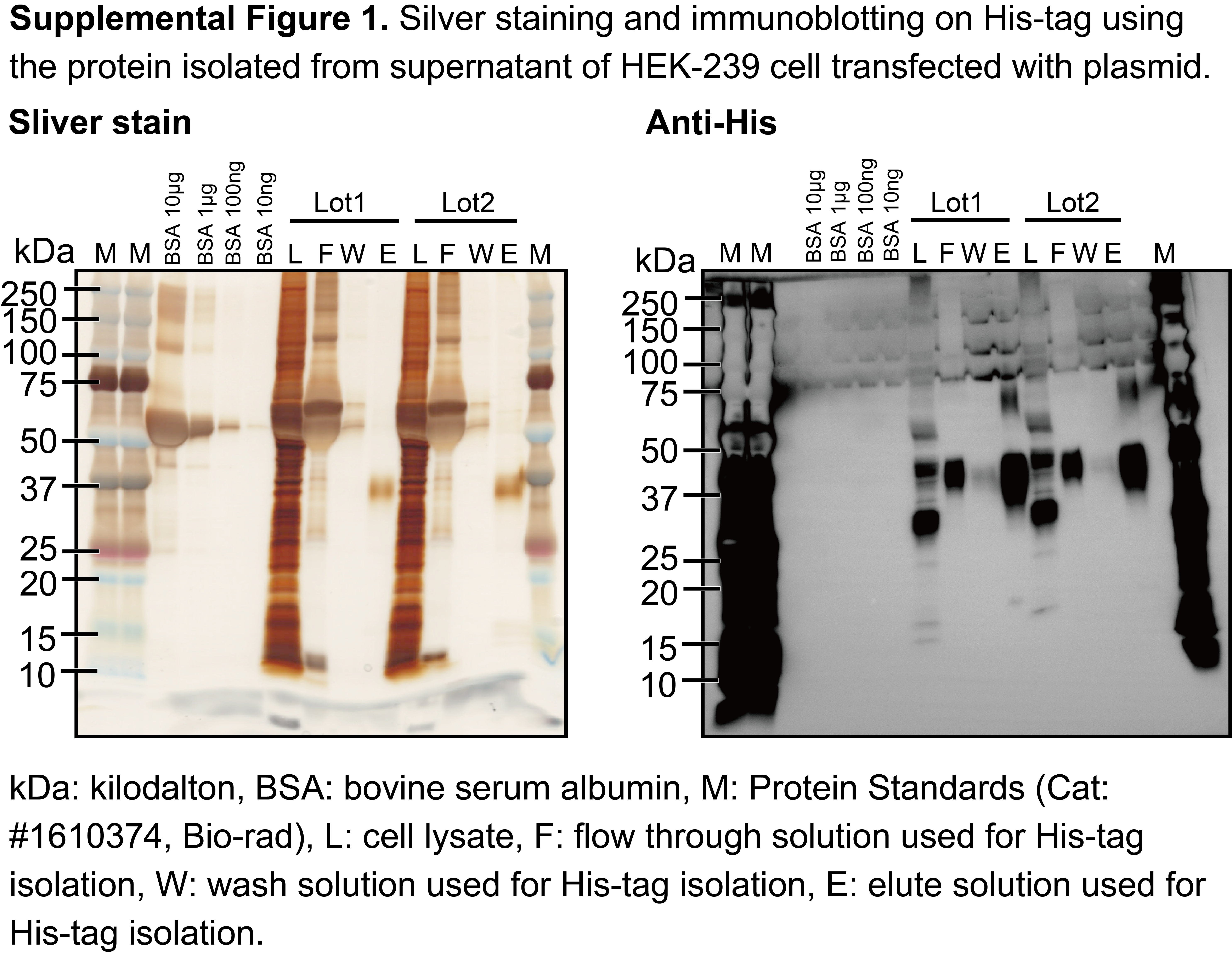

Supplement: Supplementary file 2 [file supplemental_figure.png]
